# The EVIDENT-trial: protocol and rationale of a multicenter randomized controlled trial testing the effectiveness of an online-based psychological intervention

**DOI:** 10.1186/1471-244X-13-239

**Published:** 2013-09-28

**Authors:** Jan Philipp Klein, Thomas Berger, Johanna Schröder, Christina Späth, Björn Meyer, Franz Caspar, Wolfgang Lutz, Wolfgang Greiner, Martin Hautzinger, Matthias Rose, Viola Gräfe, Fritz Hohagen, Gerhard Andersson, Eik Vettorazzi, Steffen Moritz

**Affiliations:** 1 Department of Psychiatry and Psychotherapy, Luebeck University, Luebeck, Germany; 2 Department of Clinical Psychology and Psychotherapy, University of Bern, Bern, Switzerland; 3 Department of Psychiatry and Psychotherapy, University Medical Center Hamburg-Eppendorf, Hamburg, Germany; 4 GAIA AG, Hamburg, Germany; 5 Department of Psychology, City University London, London, United Kingdom; 6 Department of Psychology, University of Trier, Trier, Germany; 7 Department of Health Economics and Health Care Management, Bielefeld University, Bielefeld, Germany; 8 Department of Psychology, Clinical Psychology and Psychotherapy, Eberhard Karls University Tuebingen, Tuebingen, Germany; 9 Department of Psychosomatic Medicine, Charité University Medical Center, Berlin, Germany; 10 Department of Behavioural Sciences and Learning, Linköping University, Stockholm, Sweden; 11 Department of Clinical Neuroscience, Karolinska Institute, Stockholm, Sweden; 12 Department of Medical Biometry and Epidemiology, University Medical Center Hamburg-Eppendorf, Hamburg, Germany

**Keywords:** Subclinical depression, Major depression, Prevention, Treatment, Online psychological treatment, Self-help, Guided self-help, Randomized controlled trial, Health economic evaluation, Cost-of-illness analysis

## Abstract

**Background:**

Depressive disorders are among the leading causes of worldwide disability with mild to moderate forms of depression being particularly common. Low-intensity treatments such as online psychological treatments may be an effective way to treat mild to moderate depressive symptoms and prevent the emergence or relapse of major depression.

**Methods/Design:**

This study is a currently recruiting multicentre parallel-groups pragmatic randomized-controlled single-blind trial. A total of 1000 participants with mild to moderate symptoms of depression from various settings including in- and outpatient services will be randomized to an online psychological treatment or care as usual (CAU). We hypothesize that the intervention will be superior to CAU in reducing depressive symptoms assessed with the Personal Health Questionnaire (PHQ-9, primary outcome measure) following the intervention (12 wks) and at follow-up (24 and 48 wks). Further outcome parameters include quality of life, use of health care resources and attitude towards online psychological treatments.

**Discussion:**

The study will yield meaningful answers to the question of whether online psychological treatment can contribute to the effective and efficient prevention and treatment of mild to moderate depression on a population level with a low barrier to entry.

**Trial registration:**

Trial Registration Number: NCT01636752

## Background

Depressive disorders are among the leading causes of worldwide disability [1]. Mild to moderate forms of depression are even more common than severe depression. While 29% of primary care patients reported mild to moderate depressive symptoms in one study only 9.5% reported moderate to severe symptoms [2]. However, subclinical forms of depression are associated with considerable impairment, economic costs, and increased risk for developing major depression [3-5].

Consequently, preventing depression and increasing access to treatment are among the most urgent global health care priorities [6,7]. Unfortunately, many individuals with depression remain untreated, even in countries with well-developed health care systems. The global mental health treatment gap has been estimated at 50% for all mental disorders [7,8]. To bridge this treatment gap and improve depression prevention and treatment, a wide array of innovative methods are needed [9]. These can be employed in a stepped-care model where the intensity of the intervention is tailored to patients’ current symptom severity [10].

Stepped-care models have been adopted in some national treatment guidelines, such as those by the National Institute of Clinical Excellence in the United Kingdom [10,11]. In such guidelines, low-intensity interventions are regarded as an appropriate first option for patients with mild to moderate depressive symptoms [10] p. 16. The spectrum of low-intensity treatments spans from self-help books to brief counseling. It includes interventions delivered by phone, mail, or SMS; or guided and unguided online psychological treatments [12]. Research on online depression treatments has garnered particular momentum in recent years, and a number of meta- analyses and systematic reviews attest to their efficacy [13-19].

Online psychological treatments have been noted to have several advantages, including easy accessibility and scalability, such that vast segments of the population can potentially be reached at low cost [20]. Not all internet interventions are easily scalable, though. Broadly speaking, the internet can be used as a communication channel as well as an information medium [20,21]. Used as a communication medium (in chat-therapy, for instance), it makes treatment more accessible for patients. However, in this form of internet-mediated intervention, therapists are still required to devote considerable time to each patient. When used as a pure information medium (i.e. unguided online self-help), treatment is both easily accessible and can be scaled up because demands on therapist time are minimized.

Meta-analyses have shown that self-guided programs, which do not require therapist support, are effective in the treatment of depressive symptoms, albeit with small effect sizes [22]. The effectiveness of unguided self-help programs can also be compromised by a high drop-out rate. In guided forms of online self-help, therapists motivate and support patients regularly, for example via brief weekly e-mail contacts. Guided online depression interventions have been shown to achieve medium to large effect sizes [13,16]. One meta-analysis even demonstrated that guided self-help can be as effective as face-to-face treatment [15]. Contacting patients prior to the onset of treatment for diagnostic purposes may increase the effect of unguided self-help programs to a medium effect size [16]. Online psychological treatments have also successfully been studied in the prevention of depressive disorders [23] and the prevention of relapse [24,25].

Even though the evidence base supporting the efficacy of online depression treatments has grown in recent years, previous studies are limited by various methodological factors. Firstly, many studies relied solely on self-reports and lacked interview-based instruments to establish diagnoses and measure symptom severity [22]. Secondly, recruitment was often carried out via advertisements or the internet rather than through clinical settings, which may introduce a bias towards more internet-savvy or motivated participants. Thirdly, most previous studies were carried out by single study sites, and the researchers were also frequently the developers of the online intervention, which may limit generalizability and foster allegiance effects. Fourthly, sample sizes in many previous studies were too small to examine moderator effects. Finally, no study to date has investigated the effect of internet-based depression treatment on subsequent help-seeking behavior. Furthermore, there is no published study known to the authors which examines the health economic consequences of internet-based treatment of depression in the German context.

We will therefore conduct a large multicenter trial to test the effectiveness of an online-based psychological treatment, compared to a care as usual control condition, among adults suffering from mild to moderate depressive symptoms, the EVIDENT trial (EffectiVeness of Internet-based DEpressioN Treatment). In this trial, mild to moderate depressive symptoms are operationalized as a score between 5 and 14 on the Patient Health Questionnaire (PHQ-9) [26]. Whereas participants with mild depressive symptoms (PHQ-9 score 5–9) will receive an unguided version of an online treatment, those with moderate symptoms (PHQ-9 score 10–14) will receive additional e-mail-support. Subjects will be recruited from a broad array of settings, including in- and outpatient medical and psychological services but also online-forums for depression, health-insurance companies and the general media (e.g., coverage in newspaper and radio reports). We will thus also be able to examine if and how online psychological treatments can be integrated into regular in- and outpatient settings.

We hypothesize that online psychological treatment will be superior to a care as usual control condition in reducing mild-to-moderate depressive symptoms. We will also analyze the effect of the intervention on the risk of developing a depressive episode in the treatment phase and in the follow-up period, which will extend to one year. We will thus be able to ascertain the effect of the intervention on the prevention of a depressive episode. Further outcome parameters include quality of life and attitude towards online psychological treatments. Moreover a cost-of-illness analysis will be conducted to determine the consumption of resources of patients with depression symptoms – separately analysed by different health care sectors.

## Methods

### Study design

**Figure 1:**
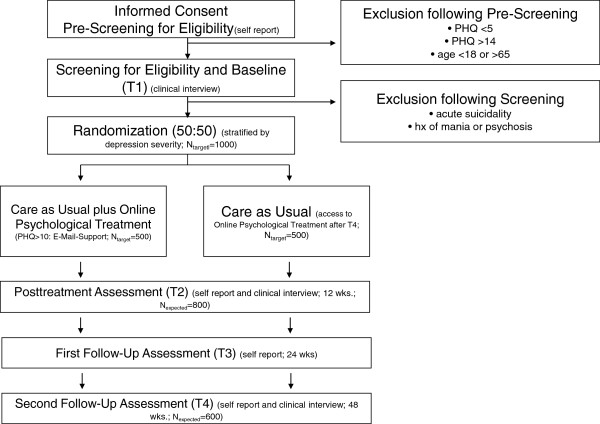
**Diagram of patient flow through the phases of study**[28]**.**

This study is a parallel-groups pragmatic randomized controlled single-blind trial. Subjects will be randomized into two groups: (1) Care-as-usual (CAU) or (2) CAU plus online-based psychological treatment. Participants in the intervention group with mild depressive symptoms will receive access to an unguided online psychological treatment, whereas those with moderate symptoms will receive the same treatment plus e-mail support (guided self-help). The study is being conducted in compliance with the Declaration of Helsinki [27]. It has been approved by the Ethics Committee of the German Psychological Association (DGPs, reference number SM 04_2012). All participants receive written information about the aim of the study, benefits and risks of participation and the study procedure. They are informed that they can withdraw at any time without having to disclose reasons. Informed consent will be obtained online prior to the baseline assessment. A written study protocol has been developed. Results will be reported in accordance with the CONSORT guidelines [28]. The study design is shown in Figure 1.

### Setting

A total of 1000 participants with symptoms of mild to moderate depression will be recruited via multiple settings, including in- and outpatient medical and psychological clinics, online-forums for depression, health-insurance companies and the media (e.g., newspaper and radio). The central recruitment tool is a study website (http://www.online-studie-depression.de), where interested persons can register for participation. Posters and postcards advertising the study are also distributed in potential recruitment sites (e.g., clinics). For the recruitment in online-forums, a standardized message advertising the study and referring to the study website will be posted in depression-specific forums by one of the coordinating centers. Participating health-insurance companies will advertise the study on their websites and in their magazines. They will also be asked to support the analysis of health care resource use described below.

### Inclusion and exclusion criteria

The main inclusion criterion for this study is the presence of mild to moderate depressive symptoms, operationalized as a score between 5 and 14 on the PHQ-9 [26]. Participants are required to be between 18and 65 years of age, have internet-access and be able to communicate in German. The main exclusion criteria are acute suicidality and a lifetime diagnosis of bipolar disorder or schizophrenia. The presence of these diagnoses will be assessed using the Mini International Neuropsychiatric Interview [MINI: 29] via telephone. Acute suicidality will be assessed clinically based on a structured assessment of current suicidal ideation and past suicide attempts [29-31]. Participants who do not meet our inclusion criteria due to severity of illness are encouraged to seek professional help.

### Randomization

Subjects will be randomized to two groups (intervention or CAU) after completion of the baseline assessment (online questionnaires and clinical interview via telephone). Stratification at one level will be performed by severity of depressive symptoms. The stratification is designed to ensure equal allocation of participants with mild depressive symptoms (PHQ-9: 5–9) and moderate depressive symptoms (PHQ-9: 10–14) to each of the two study arms. Block randomization with variable block sizes is used. An independent researcher created the allocation schedule with a computerized random number generator; other investigators are blind to this schedule. Allocation is concealed, i.e. patients and researchers have no foreknowledge of, and therefore no control over, the group to which a patient may be allocated. Subjects are informed about the randomization outcome via an email that is automatically sent to participants after the diagnostic interviewer has confirmed that inclusion- and exclusion criteria have been met. The automated email contains a code with which participants randomized to the intervention group can immediately start with the treatment. Diagnostic interviewers will be kept blind to the group assignment of the participants.

### Intervention

#### Intervention group

Participants in the intervention group receive access to a 12-week Internet-based self-help treatment (deprexis^®^) that is described in more detail elsewhere [32,33]. The self-help program consists of 10 modules, plus one introductory and one summary module. The modules cover a variety of therapeutic content that is broadly consistent with a cognitive-behavioral perspective, although the program is not restricted to one CBT manual. A specific feature of this interventionthe program is that the content of the modules is provided in simulated dialogue form, in which the program explains and illustrates concepts and techniques, engages the user in exercises, and continuously asks users for feedback. Subsequent content is then tailored to the users’ responses. Participants can go through the program at their own speed (i.e., modules are not gradually made available at a specific schedule; e.g. one module per week).

The deprexis program is available in an unguided and in a guided version [33]. The guided version will be made available to participants with an initial PHQ-9 score of 10–14. It includes a so-called therapist cockpit, where therapists/coaches can track participants’ program use and symptomatic progress. The cockpit includes an integrated and secure email system through which therapists/coaches can communicate with participants. At the beginning of the treatment, email supporters inform participants that they can contact the therapists/coaches whenever they want to. Participants are also actively contacted by their e-mail supporter once a week. In this weekly message, email supporters write short feedback based on participants’ program usage over the previous week. The feedback is relatively generic in nature and does not apply therapeutic strategies in detail. The most important aspects of this feedback are recognition and reinforcement of the participants’ independent work with the self-help program. This support will take 10–15 minutes per week, resulting in a total coaching time of about 2.5 hours per participant [33].

The email support will be provided by psychotherapists in training or Master of Science students who are in their last term of graduate programs in clinical psychology and psychotherapy. In a first step, all supporters will receive a training of approximately 4 hours in using the internet-based self-help program and on how to write the weekly feedback, based on case material. In a second step, the supporters are continuously supervised by the investigator coordinating email-support (TB). He routinely checks whether the messages written are in accordance with the treatment protocol and provides feedback to the supporters. In this multicenter study, email support is provided by three sites (Bern, Trier, Tübingen). Participants receiving email support will be randomly allocated to one of the 3 sites. However, within each site, participants will be consecutively allocated to therapists without randomization to minimize waiting times.

#### CAU group

Participants in the CAU group will not receive treatment or support by the researchers. However, they are free to seek any other help they desire, including pharmacological and psychological treatments. All concurrently used treatments will be measured repeatedly by self-reports. Participants in the CAU group will receive access to the intervention after the last follow-up assessment, 12 months after baseline assessment.

### Responses to crises and suicidality

All participants will be advised during the diagnostic interview on how to receive professional help in case of a suicidal crisis. They will be required to develop a crisis plan which includes an address of a local psychotherapist, psychiatrist, general practitioner or hospital they would contact in case of an acute crisis. In addition, an email hotline is available every weekday for the participants of the study in case of an acute crisis. This email hotline answers simple queries directly and directs participants to experts should the need arise.

### Assessments

There will be a total of four assessments. The first assessment (baseline, T1) will be before the start of the intervention. Other assessments will take place at 3 months (directly following the intervention, T2), 6 months (T3), and 12 months (T4) after baseline (see Table 1). All self-report assessments will be performed via online questionnaires conducted via an online-survey program (http://www.unipark.de). At T1, T2 and T3 an additional diagnostic interview will be conducted via telephone in which the Mini International Neuropsychiatric Interview (MINI) [29], the Hamilton Depression Rating Scale (HDRS) [30] and the Quick Inventory of Depressive Symptomatology (QIDS) [34] are administered. All clinical interviews will be conducted by certified raters. Raters are advanced university students or graduates majoring in psychology or medicine. They will all have completed training, including at least one audiotaped interview and the observation of ratings by experienced raters. In addition to the four outcome assessments, the primary outcome measure (PHQ-9) will be administered weekly in the intervention group via the online intervention and monthly for both groups during the follow-up period via a link to an online-survey that is sent via email automatically.

**Table 1:** Study assessments and assessment time points

	**Online self-assessment**									**Clinician assessment via telephone**		
	**PHQ-9**	**WSQ**	**D-CAT**	**SF-12**	**APOI**	**FEP-2**	**WAI-S**^**1,2**^	**ZUF-8**^**1**^	**Ressource use**	**HRSD**	**QIDS**	**MINI**
T1 baseline	X	X	X	X	X	X			X	X	X	X
Post randomisation	X											
T2 post-intervention	X	X	X	X	X	X	X	X	X	X	X	X
Monthly	X											
T3 three month follow-up	X	X	X	X	X	X			X			
Monthly	X											
T4 nine month follow-up	X	X	X	X		X			X	X	X	X

^1^Only patients randomized to the online psychological intervention.

^2^Only patients with moderate symptoms (PHQ-9 10–14) who also get e-mail-support.

### Outcome measurements

#### Primary outcome

##### Depressive symptoms

Symptoms of depression will be measured with the *Patient Health Questionnaire (PHQ-9)*[26]. The PHQ-9 is a self-administered version of the PRIME-MD diagnostic instrument for common mental disorders [2,26]. The PHQ-9 is the depression module, which scores at each of the 9 DSM-IV criteria on a 4-point Likert-scale from “0” (not at all) to “3” (nearly every day) [2]. The PHQ-9 score can thus range from 0 to 27. The PHQ-9 has been shown to be sensitive to change [35-38]. It can also be used as a diagnostic algorithm to make a probable diagnosis of a depressive episode [26]. The primary outcome measure (PHQ-9) will be administered once per month during the follow-up period (after the initial 12 weeks) to allow for a relatively fine-grained analysis of symptom fluctuations over time.

### Secondary outcomes

#### Diagnostic status

##### Mini International Neuropsychiatric Interview (MINI)

The MINI is a short structured diagnostic interview, developed for DSM-IV and ICD-10 psychiatric disorders [29]. In the interest of efficiency, an abbreviated version of the MINI is administered to establish the presence of the inclusion and exclusion criteria. Specifically, we assess the presence of a current depressive episode or dysthymia (Module A and B), a manic episode (Module D), a psychotic episode/schizophrenia (Module L) and acute suicidality (Module C). For the follow-up interview (T4), only the MINI Modules A and B are used to assess depressives episodes and dysthymia longitudinally. The Module A was adapted to detect the presence and onset of a depressive episode during the follow-up period. Specifically, the A5-item was modified to assess further episodes within the last 12 months (as opposed to lifetime episodes in the original version) and items A1-A4 are assessed regarding the last 12 months even in the absence of a current depressive episode (this feature is absent in the original version).

##### Web Screening Questionnaire (WSQ)

The WSQ is a 15-item self-report instrument screening for frequent mental disorders [39]. Evidence indicating adequate diagnostic validity has been reported for social phobia, panic disorder with agoraphobia, agoraphobia without panic disorder, obsessive-compulsive disorder and alcohol abuse/dependence [sensitivity .72-1.00; specificity .63-.80; 39]. Somewhat more modest psychometric properties have been reported for major depressive disorder, generalized anxiety disorder, posttraumatic stress disorder, specific phobia and panic disorder without agoraphobia [sensitivity: .80-.93; specificity: .44-.51; 39].

#### Depressive symptoms

##### Hamilton Depression Rating Scale (HDRS)

The 24-item version of the HDRS [30,40] is administered during the telephone interview (HDRS-24). It is an established clinician-rated assessment of depressive symptom severity and encompasses psychological as well as somatic symptoms (scores range from 0–2 or 0–4). Using severity descriptors, the clinician rates the severity of these symptoms based on the patient’s report, his or her own observation and third-party observations if available. Telephone-administered versions of the HDRS have been used successfully in previous studies [41]. The 24-item version is used to allow for comparisons with other depression trials [42,43].

##### Quick Inventory of Depressive Symptomatology (QIDS)

The HDRS is complemented by the Quick Inventory of Depressive Symptomatology QIDS (clinician rated). The QIDS was developed to improve on existing ratings such as the HDRS by providing equivalent weightings (0–3) for each symptom item, providing clearly stated anchors that estimate the frequency and severity of symptoms, including all DSM-IV criterion items required to diagnose a major depressive episode. The 16-item version focuses on the nine DSM-IV criterion symptom domains [43,44] and assesses the severity of depressive symptoms in the last seven days [34].

##### Computer-adaptive test for depression (D-CAT)

Computer Adaptive Tests (CAT) have been developed to enhance measurement precision and reduce respondent burden. Based on Item Response Theory (IRT), the CAT for depression (D-CAT) contains a bank of 64 depression items. These are adaptively administered until a predetermined reliability is attained. The D-CAT can be completed quickly (in just over a minute) and is reliable after an average administration of only six items [45]. The CAT-scores correlate highly with the 21-item Beck Depression Inventory (r = 0.79) and with the Centre for Epidemiological Studies Depression Scale (CES-D) 8 item short form (r = 0.76) [46]. Due to technological difficulties, the D-CAT is administered only in a subset of participants.

#### Quality of life

##### Short-Form Health Survey -12 (SF-12)

The SF-12 is based upon the “Short-Form Health Survey“ (SF-36). Its two subscales measure physical and mental aspects of health-related quality of life. It captures general health as well as pain, disabilities in daily life and mental problems. The SF-12 asks for the presence and severity of 12 items over the course of the last 4 weeks. The re-test reliability is good and it is roughly equivalent to the long form [47].

#### Health-care utilization

##### Use of health care resources

All participants are self-reporting use of health-care resources. In a subsample of patients, participants’ self-report will be compared with routine administrative data provided by several companies providing statutory health insurance. Items in the self-report cover direct (i.e. medication, inpatient and outpatient treatments) and indirect costs (i.e. days of absence due to sick leave). This will allow an estimation of direct costs (resource use) and indirect costs (productivity loss) [48,49].

#### Process evaluation

##### Working Alliance Inventory – Short (WAI-S; adapted version)

The WAI-short (WAI-S) is a 12-item self-report measure of Working Alliance [50]. Each item is rated on a 7 point scale, with higher scores indicating higher alliance. The WAI is scored into three subscales measuring Task, Goal and Bond. The Task and Goal scales are intended to measure agreement between the client and therapist with regard to Tasks and Goals of the treatment. The Bond subscale aims to measure the empathic bond between the client and the therapist [51]. We have adapted the scale by mildly reframing the items to assess internet treatment instead of face-to-face treatment. The WAI will only be administered to participants receiving guidance.

##### Patient satisfaction questionnaire (ZUF-8)

The ZUF-8 is a brief and reliable test-instrument to measure general satisfaction with treatment [52]. It was originally developed as a modified translation of the 8-item version of the Client Satisfaction Questionnaire [53] to assess satisfaction with inpatient treatment. It was adapted for this study to assess the satisfaction with the online-based psychological intervention.

#### Other outcomes

##### Questionnaire for the evaluation of psychotherapeutic progress (FEP-2)

The FEP-2 is a measure of therapeutic progress, it can be used for both change and outcome assessment [54,55]. Forty items measure the dimensions well-being, symptoms, interpersonal relationships, and incongruence with respect to approach and avoidance goals. The instrument has shown to be change sensitive as well as reliable and it is available in the public domain. It represents the phase model of therapeutic change as well as interpersonal and integrative models of psychotherapy [56].

##### Attitudes toward Psychological Online-Interventions Questionnaire (APOI)

This questionnaire will be developed based on the sample of the EVIDENT study. The development version consists of 35 items that aim at measuring attitudes towards internet-based self-help programs. After conducting exploratory and confirmatory factor analyses at the end of recruitment, the revealed latent structure of multiple factors will be applied to measure changes in participants’ attitudes toward psychological online-interventions on different dimensions. Beyond that, the aim is to identify manifestations on particular attitude dimensions that are obstructive or beneficial for the individual efficacy of internet-based self-help programs.

##### Other questions

Patients are self-reporting a number of demographic variables including sex, marital status, and level of education. They are also asked about the use of other treatments and medication, and about the referral source and internet usage.

### Sample size

The sample size is conservatively based on the expected difference between the intervention and the care-as-usual group on the primary outcome variable (i.e., depressive symptoms measured with the PHQ-9). Although the intervention evaluated in this study also includes guidance for some of the participants, the between-group effect size estimate is based on meta-analytic evidence for the effect observed in unguided psychological interventions (*d* = 0.28) [22]. Because treatments effects in a sample with mild to moderately depressed participants could even be lower, we conservatively reduced the estimated effect size to *d* = 0.23. Based on this effect size, a power of 0.80, and an alpha level of 0.05, we would need 300 subjects in each condition. Sample size was further estimated based on a drop-out rate of 40%. Drop-out rates in previous studies of the intervention used in this study range from 9% [33] to 45% [32], thus this estimate is rather conservative. Previous research shows that drop-out rates are lower in the control group [14]. Based on this assumption, we will need a total of 1000 subjects. The sample size was calculated using G-Power [57]. Based on prior experience of users of the intervention, we expect a non-eligible rate of 70% and may therefore have to screen 3333 participants.

### Statistical analysis

A statistical plan will be developed and put down in writing prior to the analysis of the data. The main analyses will be conducted on the intention-to-treat sample. A linear mixed-model repeated measures ANOVA with time (T1-T2-T3-T4) as a within groups factor and treatment condition as a between-groups factor will be used for the main research question. Mixed-model repeated measures ANOVA uses all available data of each subject and does not involve the substitution of missing values. Sensitivity analysis will be conducted to analyze the impact of drop-outs on our results. Secondary analyses of the primary endpoint will include a per-protocol approach. Per-protocol analysis will be based on full data-sets of participants who used at least two sessions of the online-based psychological intervention for a complete duration of at least 60 minutes. The analysis of time to onset of a depressive episode and time to remission of depressive episode will be analyzed using survival analysis techniques. The time to onset of depressive episode will be based on monthly PHQ-9 assessments (these allow the assessment of a probable presence or absence of a depressive episode [26]) and retrospective assessment of the onset of MDE during the final telephone interview (T4). Significance testing of dichotomous data such as diagnostic status will be conducted with chi-square tests. Calculations of within- and between-groups effect sizes (Cohen’s d) will be based on the pooled standard deviations. Regression analyses will be used to identify predictors of treatment outcome. To analyse the use of health-care resources descriptive methods such as absolute and relative frequency scales, measures of dispersion or measures of central tendency will be used. In order to investigate differences between various patient subgroups appropriate statistical tests will be applied. The assortment of the test will depend on the character of the routine administrative data. All analyses will be performed with SPSS statistics, Excel and Visual Basic for Applications (VBA).

## Discussion

This randomized-controlled multicenter study examines the effectiveness of an online psychological intervention in controlling mild to moderate depressive symptoms and preventing emergence and relapse of depression. It is one of the first large trials examining the impact of online self-help interventions on health economic measures [58]. We will also gain valuable experiences in implementing an online psychological intervention into routine clinical care.

As a major strength of this study, it has a low-threshold of entry as anyone suffering from depressive symptoms can sign up through the study website without further requirements. We will therefore be able to draw inferences with regard to the effectiveness of online-psychological treatments, i.e. the extent to which the treatment achieves its intended effect in the usual clinical setting [59]. To this end, we will include patients with other comorbid disorders including substance abuse and dependence, anxiety disorders and personality disorders. Also, acute suicidality will be assessed clinically by trained interviewers rather than based on a more rigid exclusion of all patients who cross a certain threshold on a self-rating. These measures will improve the external validity of our study results as the great majority of patients with depression suffer from at least one comorbid mental disorder (41). This strength of the study may also be regarded as a limitation, as it may result in a relatively heterogeneous group of patients. Another weakness of our design is the liberal acceptance of concomitant treatments in our study, which again will increase the external validity of our findings while possibly reducing internal validity.

In addition to the main analysis described in the methods section, our study will be able to address a number of other important research questions, some of which have not been addressed before. To this end, we will conduct subgroup analyses of the influence of pre-specified baseline characteristics on the main outcome measure [60] and analyses of secondary outcomes. One subgroup analysis will concern the influence of referral source (medical and psychological services versus other) and primary motivation for study participation (self versus other) on the main outcome as many trials of online-based psychological interventions have been conducted outside routine clinical practice [22] and the applicability of the results to routine clinical practice has therefore been debated [61]. Other subgroup analysis will concern the influence of baseline severity [14], presence of depressive episode at baseline, chronicity [62], parallel treatment [24] and attitude to online psychological intervention [63] on the main outcome.

We also plan to undertake a wide range of additional analyses. Weekly PHQ assessments in the intervention group will allow us to identify meaningful patterns of early change in depression during online treatment that are shared by many individual patients and examine whether these patterns predict outcomes at treatment termination and over the follow-up period as well as drop-out or time patients participate in online treatment [64,65]. Also we may be able to assess whether e-mail support affects working alliance scores and attitudes towards online psychological interventions. The results of this analysis will be biased by depression severity however as only more severely depressed participants receive e-mail support. Finally, this study will be the first to use the Computer-adaptive test for depression (D-CAT) [45,46] in a prospective randomized controlled trial and will allow the comparison with more established outcome measures.

The present trial will probably be the largest multicentric study conducted thus far of an online depression treatment in which validated diagnostic interviews and symptom severity measures are used and participants are followed for a full year (without those in the control group receiving the intervention). It is hoped that the study will yield meaningful answers to the question of whether the internet-based intervention studied here can contribute to the effective and efficient prevention and treatment of mild to moderate depression on a population level. The results of this trial are expected to influence policy decisions with regard to whether such interventions ought to be implemented more widely in order to meet the challenge posed by the global depression treatment gap.

## Trial status

Currently recruiting (N_current_ = 796 as of September 5^th^ 2013).

## Abbreviations

APOI: Attitudes toward psychological online-interventions questionnaire; CAU: Care-as-usual; CES-D: Centre for epidemiological studies depression scale; D-CAT: Computer-adaptive test for depression; FEP-2: Questionnaire for the evaluation of psychotherapeutic progress (Fragebogen zur evaluation von psychotherapieverläufen); HDRS: Hamilton depression rating scale; MINI: Mini international neuropsychiatric interview; PHQ-9: ; QIDS: Quick inventory of depressive symptomatology; SF-12: Short-form health survey -12; WAI-S: Working alliance inventory – Short form; WSQ: Web screening questionnaire; ZUF-8: Patient satisfaction questionnaire (Fragebogen zur Patientenzufriedenheit).

## Competing interests

BM is employed as research director at GAIA AG, the company that developed and owns the internet-based psychological intervention investigated in this trial.

## Authors’ contributions

JPK participated in the conception and design of the study, the acquisition of data and drafted the first manuscript. TB participated in the conception and design of the study and in drafting the manuscript and data acquisition. JS participated in the design of the study and in drafting the manuscript and data acquisition. CS participated in drafting the manuscript and data acquisition. BM participated in the conception and design of the study and in drafting the manuscript. FC participated in the design of the study. WL participated in the design of the study and in drafting the manuscript and data acquisition. WG participated in the design of the study and in drafting the manuscript and data acquisition. MH participated in the design of the study and in drafting the manuscript and data acquisition. MR participated in the design of the study and in data acquisition. VG participated in drafting the manuscript and data acquisition. FH participated in the conception and design of the study and in data acquisition. GA participated in design of the study and in drafting of the manuscript. EV participated in design of the study and in drafting of the manuscript. SM participated in the conception and design of the study, the acquisition of data and in drafting the manuscript. All authors read and approved the final manuscript.

## Pre-publication history

The pre-publication history for this paper can be accessed here:

http://www.biomedcentral.com/1471-244X/13/239/prepub
